# Optimization of Exercise Countermeasures for Human Space Flight: Operational Considerations for Concurrent Strength and Aerobic Training

**DOI:** 10.3389/fphys.2019.00584

**Published:** 2019-05-16

**Authors:** Thomas W. Jones, Nora Petersen, Glyn Howatson

**Affiliations:** ^1^ Department of Sport, Exercise and Rehabilitation, Northumbria University, Newcastle upon Tyne, United Kingdom; ^2^ KBRwyle GmbH, European Astronaut Centre, European Space Agency, Cologne, Germany; ^3^ Space Medicine Team, European Astronaut Centre, European Space Agency, Cologne, Germany; ^4^ Water Research Group, School of Environmental Sciences and Development, North West University, Potchefstroom, South Africa

**Keywords:** microgravity, strength training, aerobic training, exercise recovery, neuromuscular adaptation

## Abstract

The physiological challenges presented by space flight and in microgravity (μG) environments are well documented. μG environments can result in declines muscle mass, contractile strength, and functional capabilities. Previous work has focused on exercise countermeasures designed to attenuate the negative effects of μG on skeletal muscle structure, function, and contractile strength and aerobic fitness parameters. Exposure to μG environments influences both strength and aerobic type physical qualities. As such, the current exercise recommendations for those experiencing μG involve a combination of strength and aerobic training or “concurrent training.” Concurrent training strategies can result in development and maintenance of both strength and aerobic capabilities. However, terrestrial research has indicated that if concurrent training strategies are implemented inappropriately, strength development can be inhibited. Previous work has also demonstrated that the aforementioned inhibition of strength development is dependent on the frequency of aerobic training, modality of aerobic training, the relief period between strength and aerobic training, and the intra-session sequencing of strength and aerobic training. While time constraints and feasibility are important considerations for exercise strategies in μG, certain considerations could be made when prescribing concurrent strength and aerobic training to those experiencing human space flight. If strength and aerobic exercise must be performed in close proximity, strength should precede aerobic stimulus. Eccentric strength training methods should be considered to increase mechanical load and reduce metabolic cost. For aerobic capacity, maintenance cycle and/or rowing-based high-intensity intermittent training (HIIT) should be considered and cycle ergometry and/or rowing may be preferable to treadmill running.

## Exercise Countermeasures During Human Space Flight and Microgravity

Human space flight and microgravity (μG) environments present numerous physical challenges (Petersen et al., 2016). A common, yet troublesome symptom of μG environments is the decline in skeletal muscle mass and strength (Riley et al., 2000; Fitts et al., 2010). This decline is attributable to μG rendering the body and other objects weightless, negating the requirement for muscular contractile forces to elicit movement of the body or external objects (Lackner and DiZio, 1996). Strength qualities are important for situations such as emergency egress, in flight maneuvers and returning to weight-bearing terrestrial environments (Laughlin et al., 2015). In addition, aerobic capabilities are required to sustain functional capacities and conduct activities such as prolonged space walks (Hackney et al., 2015; Hayes, 2015). As such, strategies are needed to maintain both strength and aerobic type physical qualities in μG environments.

Early work examined the effects of resistance exercise strategies on the maintenance of muscle integrity in crew members experiencing imposed bed rest. It was reported that strength training methods resulted in maintenance of muscle integrity during prolonged periods of bed rest (Brannon et al., 1963). These findings lead to subsequent work on exercise in actual or simulated μG, rather than using bed rest as a proxy (Zamparo et al., 1992; Murthy et al., 1994). Initially, exercise strategies for those experiencing μG involved a combination of continuous aerobic exercise *via* loaded cycling and running and walking on a treadmill with numerous bungee cords and restraints. Rudimentary strength training strategies using bungee cords were also prescribed (Kozlovskaya et al., 1995). While it was reported that such strategies could promote maintenance of muscle quality, subsequent work demonstrated that this may not always be the case. Trappe et al. (2009) documented the exercise program undertaken by crew members aboard the International Space Station (ISS) and examined its effectiveness for preserving calf muscle characteristics. It was reported that during the 6-month period, crew members engaged in ~5 days week^−1^ of moderate aerobic exercise and 3–6 days week^−1^ of resistance training. After 6 months, crew members experienced reduction in calf muscle mass and performance, indicating that the strength and aerobic stimuli reported here were insufficient to maintain muscle integrity. Following this work, it was proposed that future long duration space missions should modify exercise prescription. Subsequently, the Integrated Resistance and Aerobic Training protocol (SPRINT) was constructed. The SPRINT protocol was based on previous work into exercise and muscle fiber function in those experiencing bed rest (Trappe et al., 2004, 2008). The SPRINT protocol is notably different to the previous μG exercise prescriptions and is characterized by alternative days of strength and continuous aerobic exercise and interval type aerobic exercise (Murach et al., 2018). It is apparent that those preparing for and experiencing human space flight are required to train concurrently for the development and maintenance of strength and aerobic qualities. Furthermore, if these concurrent training strategies are to be delivered efficiently to maximize strength and aerobic development, certain program variables need to be considered.

## Concurrent Strength and Endurance Training and its “Interference Effect”

Training for the maintenance and/or development of both strength and aerobic physical qualities is termed “concurrent training” and has been associated with suboptimal strength adaptations when compared with strength training performed in isolation (Hickson, 1980; Methenitis, 2018; Murlasits et al., 2018). While combining strength and aerobic training does not appear problematic in untrained populations, challenges are presented in well-trained individuals such as crew members. Previous work has reported crew members to possess good strength capabilities (lower body power = ~2,000 W, upper body power = ~1,000 W, lower body maximum isometric force ~2,200 N, and vertical jump = ~40 cm) (Loehr et al., 2011; English et al., 2015; Mulavara et al., 2018). In addition, it has been reported that crew members also exhibit heightened aerobic capabilities (aerobic capacity index = ~4.0 L min^−1^ for males and ~2.5 L min^−1^ for females) (Moore et al., 2015).

The muted strength development associated with concurrent strength and aerobic training is termed “interference effect” (Hickson, 1980). Combining strength and aerobic training is potentially challenging as there is requirement for crew members to train concurrently in μG environments in order to maintain muscle mass and contractile strength. This presents a potential issue for the crew members operating in μG environments where the strength training stimulus can be compromised by the aerobic type stimulus. Crew members have limited time to perform exercise regimens; therefore, it is essential that any exercise training performed has a positive influence on the individual’s functional capabilities. As concurrent training might elicit suboptimal strength adaptations, it is reasonable to suggest that concurrent training is not as effective as separated training sessions, and may not always be appropriate for crew members seeking to maintain physical performance in μG. However, in the reality of space flight, most daily exercise sessions are preferably performed within the same session by crew members, in order to remain time efficient for their duties on board the space station.

**Figure 1:**
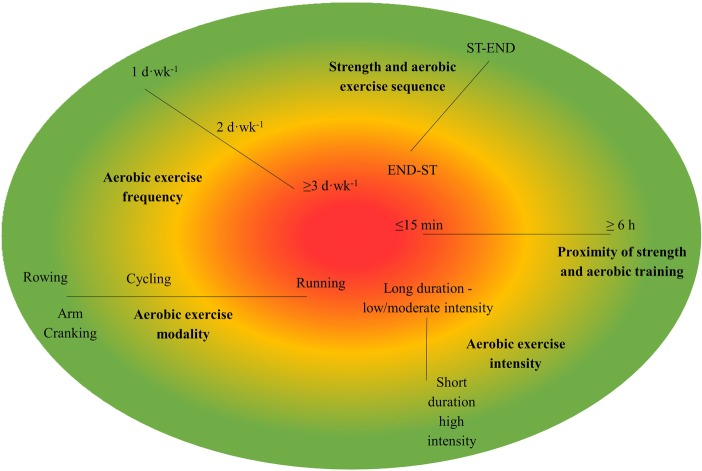
Heat map of program variables and their contributions to the interference effect. Green, not likely to result in interference; amber, possible to result in interference; red, likely to result in interference; ST-END, strength before endurance training; END-ST, endurance before strength training.

Previous work investigating concurrent training and the interference effect has indicated that the presence and magnitude of any blunted strength adaptations are influenced by program variables including volume, frequency, and modality of aerobic training and the order of strength and aerobic stimulus (Wilson et al., 2012; Jones et al., 2013, 2016; Eddens et al., 2018). Figure 1 summarizes how program variables can contribute to interference characteristics.

It is also worth noting that a large body of evidence has demonstrated that strength training can positively affect endurance performance. Augmented performance is typically *via* improvements in economy/efficiency (Mikkola et al., 2007; Barnes et al., 2013; Barnes and Kilding, 2015). Conversely, a recent review has suggested that if aerobic training is repeatedly performed under strength training-induced residual fatigue, aerobic type performance may be compromised (Doma et al., 2019). While this may be problematic, it is reasonable to suggest that if a sufficient recovery period is permitted between training modalities, any potential negative effect of strength training on aerobic adaptation may be avoided.

## Program Variables

### Training Volume

The volume and frequency of aerobic training performed within a concurrent training regimen may be key program variables that influence any impaired strength development and/or maintenance (Wilson et al., 2012; Jones et al., 2013, 2016), volume being the total time of aerobic training and frequency being the number of aerobic sessions. Higher volumes of aerobic training tend to result in more pronounced inhibition of strength development. Research employing differing ratios of strength and aerobic training reported strength development to be similar in those who performed strength training alone and concurrent training at a ratio of 3:1 in favor of strength training (Jones et al., 2013, 2016). By contrast, those who performed equal frequencies of strength and aerobic training (three sessions per week of both strength and aerobic training) experienced smaller increases in maximal strength than those who performed strength training alone and those who performed three strength sessions per week and one aerobic session (ratio of 3:1). This impact of aerobic training frequency on strength development was observed following both isolated limb (Jones et al., 2013) and multi-joint (Jones et al., 2016) training interventions. Following the multi-joint intervention, those who performed three strength and three aerobic sessions per week not only experienced impaired strength development but also elevations in basal cortisol levels (Jones et al., 2016). It is reasonable to suggest that the muted strength development following higher frequencies and volumes of strength and aerobic training may be attributable to elevated physical stress.

A meta-analysis has indicated that longer durations of aerobic stimulus can result in greater inhibition of strength development (Wilson et al., 2012). However, there is a caveat to this fact. Previous work has reported that the soleus muscle is highly susceptible to unloading due to its oxidative nature (Gallagher et al., 2005; Trappe et al., 2008); it was also reported that to maintain integrity of the soleus muscle, higher volumes of stimulation are required. As such, it appears that the role of volume in interference and the maintenance of physical qualities may be specific to individual muscle groups.

The current guidelines for exercise during human space flight involve concurrent strength and aerobic training, with strength training contributing to 54% of the total training volume for European Space Agency (ESA) Crews (Petersen et al., 2016). However, these relative contributions may depend on the individual training protocol and also may vary between crew members of different space agencies. Separate work has also indicated that these concurrent strategies are high in frequency, with two (one strength and one aerobic) sessions a day being performed 6 days week^−1^ (Loehr et al., 2015). Combined, this may indicate that the current situation on ISS of predominantly concurrent training during long duration missions might result in impaired maintenance of contractile strength and muscle function. Despite the current prescriptions, it is reasonable to suggest that, from a strength perspective, crew members might benefit from reducing the volume of aerobic training in order to provide adequate stimulus to attenuate losses in aerobic capacity and maintain strength. The benefits of this would allow physical qualities to be maintained and reduce the need to consume increased oxygen to train aerobically and of course reduce the consequent production of CO_2_; as a consequence, the burden of regulating ambient CO_2_ levels would be reduced.

### Training Modalities

Aboard ISS, the use of two devices for aerobic training (treadmill and cycle ergometer) might seem appropriate and provide a variety of aerobic stimuli. It is not only aerobic exercise volume/frequency that impacts on the magnitude of the interference effect. A meta-analysis has indicated the modality of aerobic exercise stimulus influences interference characteristics (Wilson et al., 2012). The meta-analysis of 21 studies reported running to negatively impact on strength development but not cycling (Wilson et al., 2012). As such, it is logical that in μG environments, cycle ergometry would be more conducive to maintaining strength than treadmill running. Furthermore, there is no evidence to suggest that continuous or intermittent rowing results in any inference characteristics. In fact, recent work has indicated that resistance training using a gravity-independent flywheel and aerobic training *via* continuous and intermittent rowing was able to preserve several key muscle characteristics during 70 days of bed rest (Murach et al., 2018). It should also be noted that Murach et al. (2018) observed a combination of eccentric strength training and intermittent rowing successfully preserved muscular integrity of the soleus.

As previously stated, the soleus responds differently to other muscle groups to μG and exercise exposures (Gallagher et al., 2005; Trappe et al., 2008). It is possible that mechanical loading-based aerobic exercise like running can better maintain the qualities of the soleus in μG. An additional meta-analysis has indicated that if running is performed as high-intensity intermittent training (HIIT), then inference characteristics can be avoided (Sabag et al., 2018). As such, in some cases, HIIT running may be a viable aerobic training strategy.

The current time allocation for exercise aboard the ISS is 2.5 h day^−1^. However, in future human exploration missions, it is possible that greater restrictions will be placed on exercise time. If this is the case, it is imperative that any exercise performed does not inhibit the maintenance of other physical qualities. Irrespective of whether exercise time on long duration missions is reduced, to maintain physical health and functionality, crew members will still be required to perform strength and aerobic training. If time available to exercise is reduced, the nature of the training, would of course, needs to be streamlined. From a strength perspective, the implementation of eccentric training methods (given that the injury risk remains low) may be beneficial. Eccentric muscle actions have the potential to produce high forces (when compared with concentric contractions) with low metabolic costs (McHugh et al., 2002) and hence reduce the increase oxygen cost. Furthermore, the nature of eccentric training methods could be well suited to μG environments. Data pooled from nine studies have indicated that iso-inertial flywheel resistance training involving eccentric overload triggers greater skeletal muscle adaptations (strength, power, and muscle mass) compared to gravity-dependent resistance training paradigms (Maroto-Izquierdo et al., 2017). As eccentric training elicits higher forces with greater skeletal muscle adaptation, it could be argued that maintenance of strength type qualities in μG environments could be achieved more efficiently with eccentric strength training methods. Furthermore, there is no evidence that eccentric strength training methods combined with aerobic training results in muted strength development. Concurrent training studies involving primarily or ideally exclusively eccentric training methods would provide useable inferences for practitioners supporting crew members who experience μG environments. In addition, there is also evidence that acute eccentric training improves mitochondrial calcium homeostasis and may stabilize mitochondrial respiratory function (Rattray et al., 2013). This could suggest that concurrent training with eccentric strength training may have additional benefits.

Currently, during long duration missions, crew members perform a combination of steady-state and interval aerobic training (Loehr et al., 2015). If time available to exercise is reduced, HIIT strategies could be considered. Previous work has indicated that HIIT can be equally effective as continuous training for improving aerobic capacity, despite HIIT duration and volume being much lower than that of continuous training (Tabata et al., 1996; Gibala et al., 2006). Furthermore, when matched for total volume, HIIT has been reported to improve aerobic capacity to a greater extent that moderate-intensity training (Helgerud et al., 2007). Not only does HIIT appear to be a viable option for aerobic exercise under greater time constraints, there is also no evidence that short duration HIIT results in impaired strength responses in concurrent training regimens.

### Scheduling of Strength and Aerobic Training

In addition to frequency, volume, and modality of aerobic type stimulus, the order in which strength and aerobic training are performed can also influence the adaptations to concurrent training. A recent meta-analysis examined whether intra-session concurrent exercise sequence modulates strength-based outcomes associated with the interference effect (Eddens et al., 2018). The analysis indicated that strength followed by aerobic exercise is more favorable for improving dynamic strength than *vice versa*. It is likely that this is due to strength training being more effective when performed in a non-fatigued state (i.e., not following prior aerobic exercise) (Sporer and Wenger, 2003). In addition, it is unlikely that aerobic training involving the lower body musculature impacts upper body strength (Jones et al., 2016). Due to the nature of human space flight and long duration missions, it is inevitable that any exercise performed will be placed under strict time constraints. It has been reported that during human space flight, strength and aerobic training can take place “back-to-back” or in close proximity with minimal relief period between training modalities (Petersen et al., 2016). Previous work has indicated that the relief period between strength and aerobic training (even when strength is conducted prior to aerobic training) can influence both acute and chronic strength performance and adaptations (Docherty and Sporer, 2000; Sporer and Wenger, 2003; Robineau et al., 2016). Previous work has indicated that strength and aerobic stimuli should be separated by ≥6 h if impairments in strength development are to be avoided (García-Pallarés and Izquierdo, 2011; Robineau et al., 2016). This suggestion is supported by recent work indicating that combined strength and continuous and intermittent rowing conducted with a 4-h relief period resulted in maintenance of muscle characteristics during 70 days of bed rest (Murach et al., 2018). These data indicate that if time constraints are a concern, a 4-h relief period between strength and aerobic stimuli is permissible. It is also perhaps reasonable to suggest that aerobic training primarily involving the lower body musculature (e.g., cycling) could be followed by upper body strength training.

## Recommendations for Programming and Future Research

To conclude, it appears that the volume, frequency, order, and modality of strength and aerobic training can influence the responses to combined strength and aerobic training. Based on what is known about combining strength and aerobic training in terrestrial environments, evidence-based programming recommendations can be made regarding concurrent training in μG. It should however be noted that these are general recommendations and may not necessarily apply to all muscle groups.

The aim of these recommendations is to promote maintenance of strength and aerobic qualities, while minimizing the potential confounding factors associated with concurrent training:

- If strength and aerobic exercise must be performed in close proximity, strength should precede the aerobic stimulus. However, if possible, a 4-h relief period should be permitted.
- For aerobic capacity maintenance, cycle ergometry and/or rowing may be preferable to treadmill running.
- Eccentric strength training methods should be considered to increase mechanical load and reduce metabolic cost.
- For aerobic capacity, maintenance cycle and/or rowing-based HIIT should be considered.

The effects of differing concurrent training strategies in μG environments have not been investigated, and there are limited opportunities to perform controlled intervention studies in a μG environment. This is because flight rules and medical requirements on the ISS stipulate that crewmembers must conduct exercise strategies designed to counteract the negative effects of μG (and no crew wants to take the risk of serious deconditioning). Therefore, the opportunities to manipulate program variables and have individuals performing different exercise strategies within the same long-duration mission are limited. As such, presently we must refer to what we know about combining strength and aerobic training in terrestrial environments and apply these to μG paradigms. It is also not unreasonable to speculate that the elevated physical stress associated with μG environments may result in a more pronounced interference effect. As such, future work should perhaps compare the current exercise prescription of exercise in μG against a concurrent strength and aerobic regimen specifically designed to avoid any interference characteristics.

Future research into exercise countermeasure for human space flight may seek to address the following questions:

- Do high volumes of aerobic training result in inhibited strength development/maintenance in μG?
- Do frequency, order, and modality of strength and aerobic training influence any inhibited strength development/maintenance in μG?
- Are eccentric training methods compatible with aerobic training in μG environments?

## Author Contributions

TJ and GH wrote the initial drafts with NP reviewing drafts.

### Conflict of Interest Statement

The authors declare that the research was conducted in the absence of any commercial or financial relationships that could be construed as a potential conflict of interest.
